# Supplementary material for: Timing of emergence of modern rates of sea-level rise by 1863
Source: Nat Commun. 2022 Feb 18;13:966. doi: 10.1038/s41467-022-28564-6 (PMC8857177; doi:10.1038/s41467-022-28564-6)
Supplement: Supplementary file 1 — Supplementary Information [file 41467_2022_28564_MOESM1_ESM.pdf]

Timing of emergence of modern rates of sea-level rise by 1863

Supplementary Figures

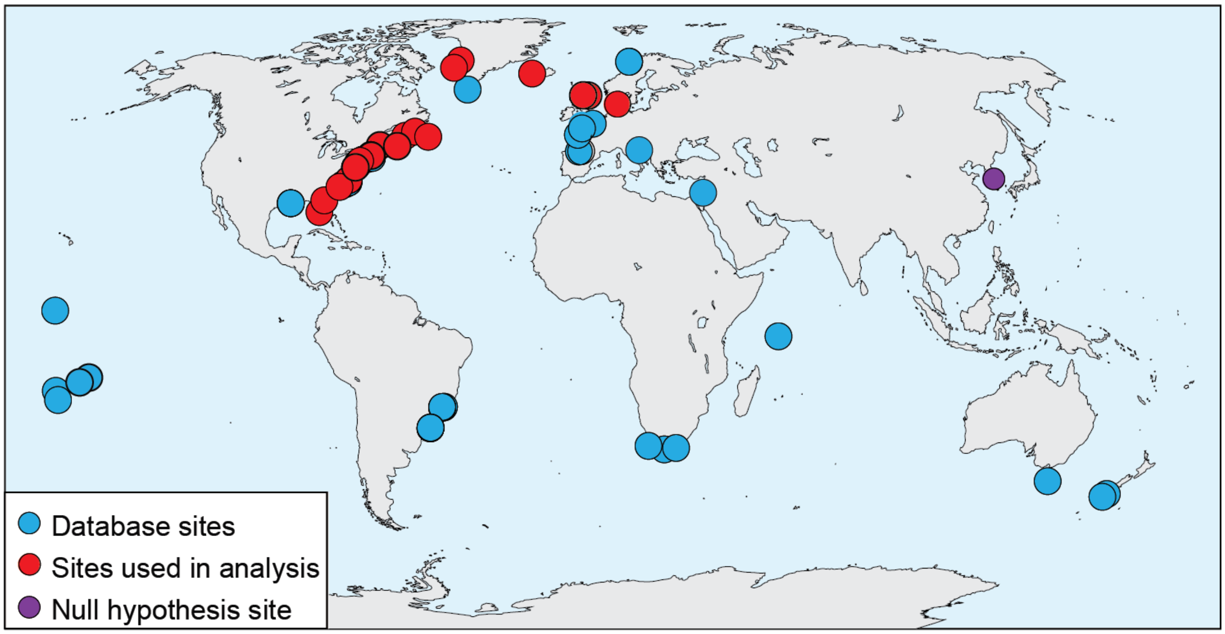

Supplementary Figure 1. Proxy sea-level records in the Common Era sea-level database. Sites used in time of emergence analysis are shown in red and null hypothesis site is shown in purple.

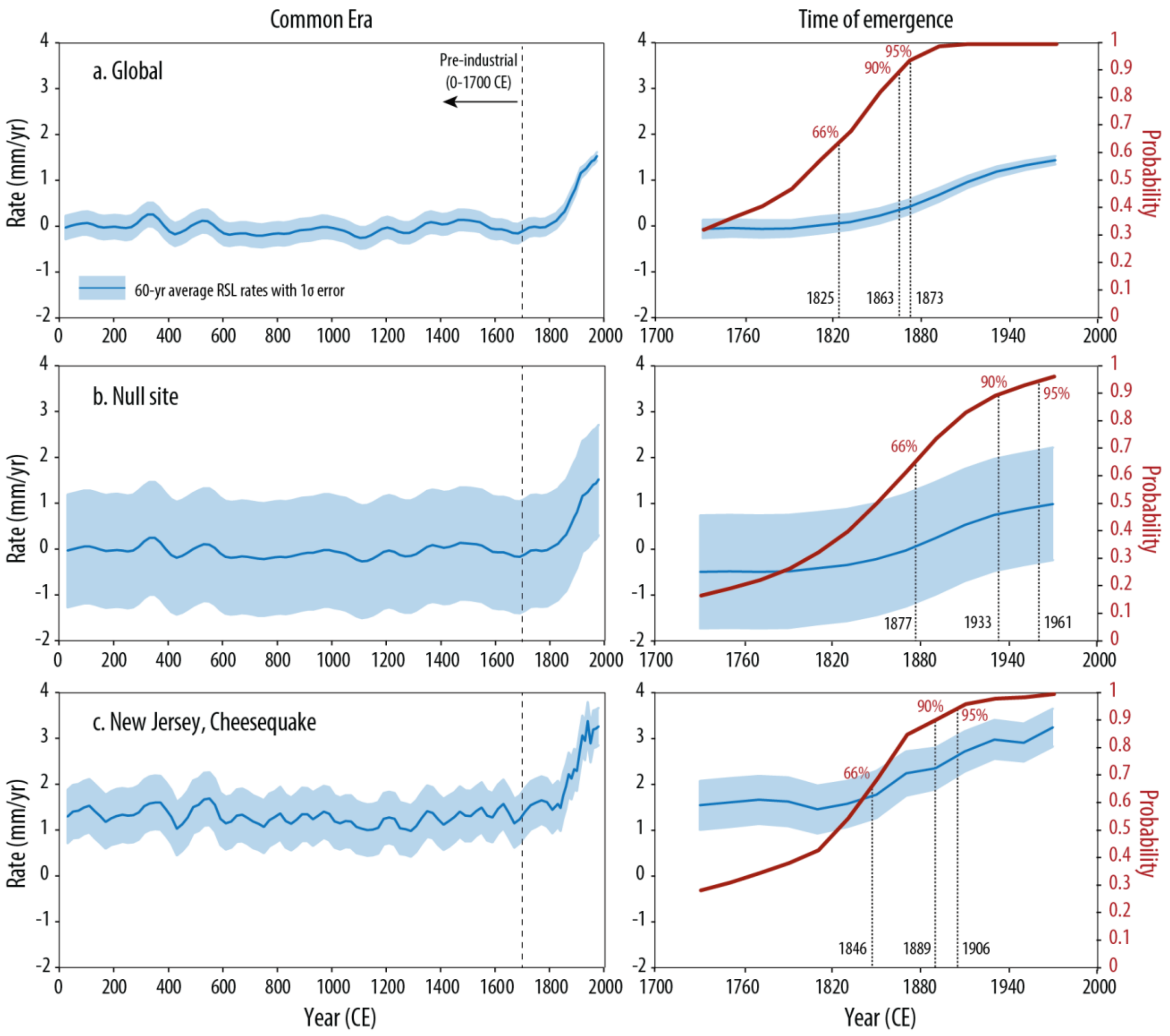

Supplementary Figure 2. Global sea level, null site, and New Jersey time of emergence. Sixty-year average rates over the Common Era for (a) global sea level, (b) null site, and (c) New Jersey (Cheesequake). The time of emergence (ToE) year is given for 0.66, 0.90, and 0.95 probabilities. Model predictions are the mean with 1σ uncertainty. The North Atlantic sites have unique RSL predictions and ToE estimates that are more precisely constrained than under the null hypothesis that local RSL is equal to global sea level plus variability, reflecting the influence of meaningful constraints from proxy data.

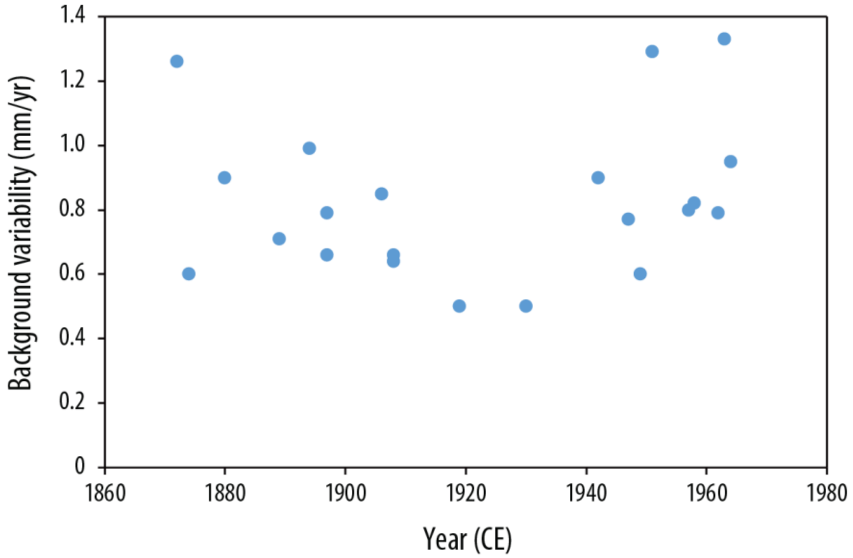

Supplementary Figure 3. Pre-industrial background variability versus time of emergence. The background variability is the distribution of mean estimates of background rates from 0–1700 CE where the value is the highest minus the lowest background rate. There is no correlation at the North Atlantic sites between distributions of mean estimates of background rates and ToE. In fact, some of the sites with the greatest pre-industrial range of rates have some of the earliest ToE, implying regional processes rather than background variability driving ToE.

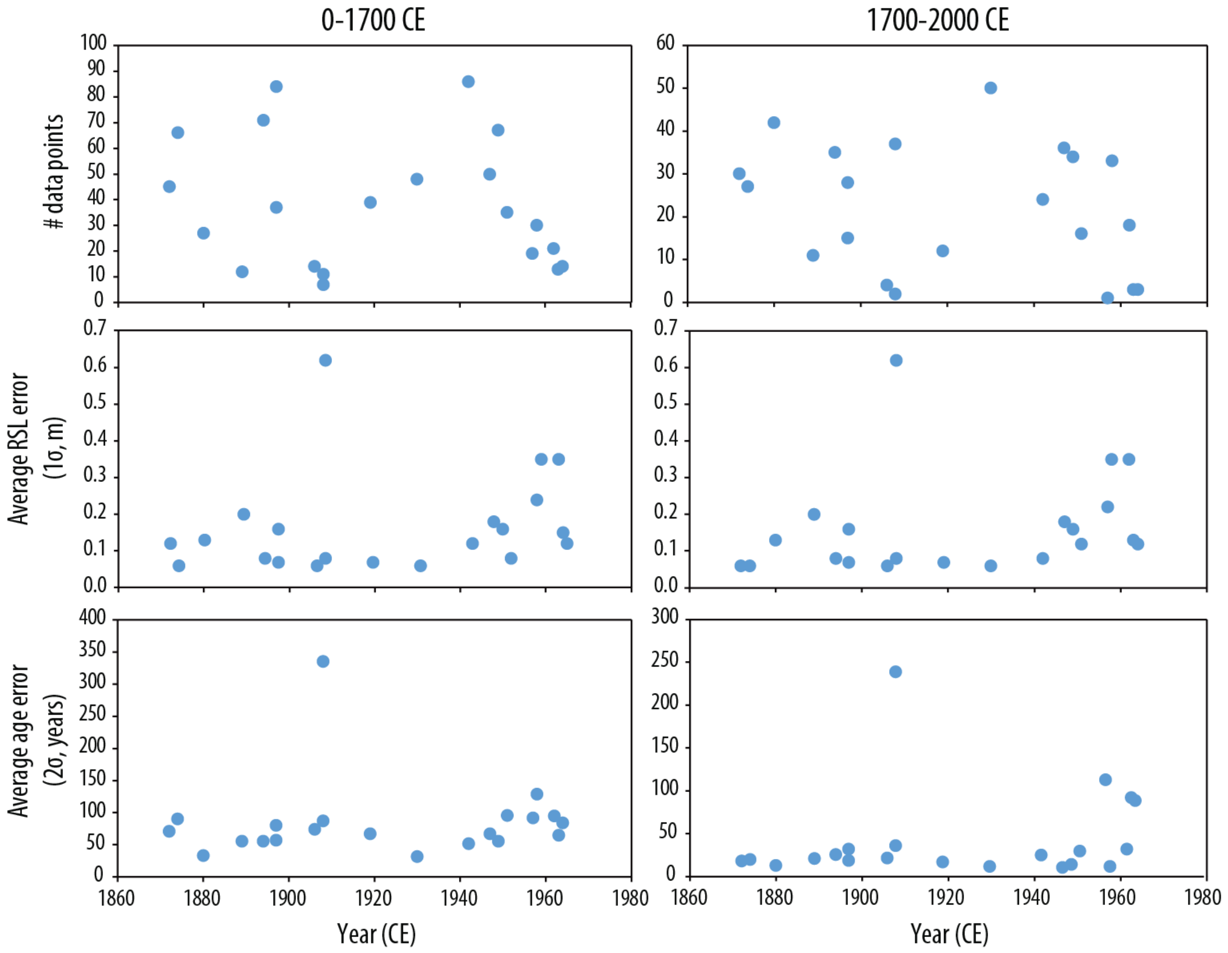

Supplementary Figure 4. Proxy data versus time of emergence. Relative sea level proxy data information over 0–1700 and 1700–2000 CE for 21 North Atlantic sites (see Supplementary Data Table 2) compared to each site's time of emergence.

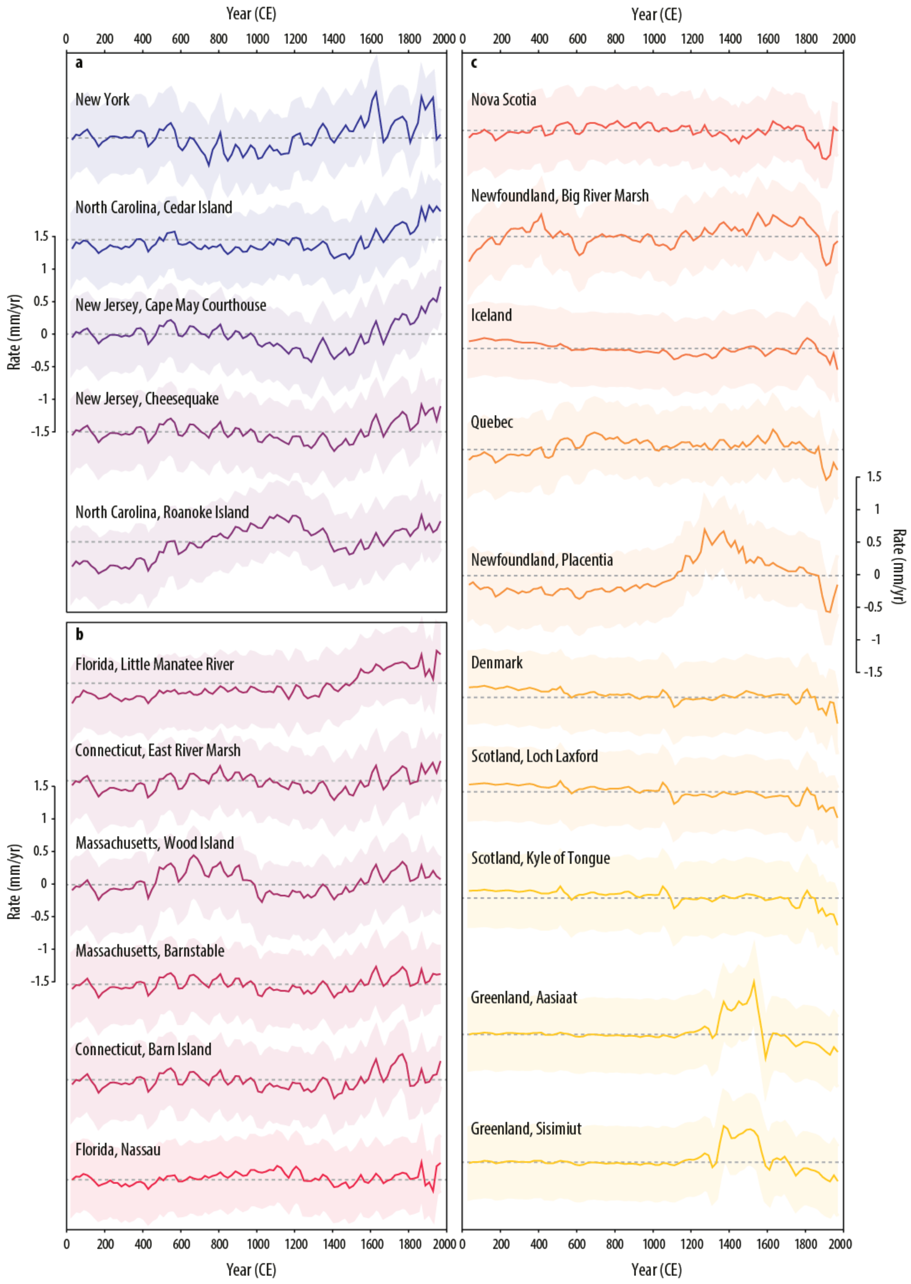

Supplementary Figure 5. Combined regional and local nonlinear rates of Common Era relative sea level. The global and linear components have been removed. Rates are shown for (a) mid-Atlantic U.S.; (b) northeastern and southeastern U.S.; and (c) Canada and Europe sites, which are color coded by time of emergence according to Figure 2. Model predictions are the mean with 1σ uncertainty.

Supplementary Tables

Supplementary Table 1. Optimized hyperparameters for spatiotemporal statistical model.

Term	Prior SD	Characteristic timescale (years)	Characteristic length scale (degrees)
$g_f(t)$ [fast global]	±0.2 cm	12	-
$g_s(t)$ [slow global]	±13 cm	355	-
$m(\mathbf{x})$ [linear]	±1.1 mm/yr	-	3.6
$r_f(\mathbf{x},t)$ [fast regional]	±2 cm	12	12.0
$r_s(\mathbf{x},t)$ [slow regional]	±3 cm	355	12.0
$l_f(\mathbf{x},t)$ [fast local]	±2 cm	12	0.04
$l_s(\mathbf{x},t)$ [slow local]	±3 cm	355	0.04
$w(\mathbf{x},t)$ [additional error]	±0.03 mm	-	-
$y_0(\mathbf{x})$ [datum offset]	±0.1 mm	-	-

Supplementary Table 2. Relative sea level proxy data information for 21 North Atlantic sites used in time of emergence analysis.

Site with reference	# data points (0–1700 CE)	# data points (1700–2000 CE)	Average RSL error, 1σ, (0–1700 CE)	Average age error, 2σ, (0–1700 CE)	Average RSL error, 1σ, (1700–2000 CE)	Average age error, 2σ, (1700–2000 CE)
New York, Pelham Bay[1]	45	30	0.12	71	0.06	18
North Carolina, Cedar Island[2,3]	66	27	0.06	90	0.06	20
New Jersey, Cape May Courthouse[4,5]	27	42	0.13	33	0.13	13
New Jersey, Cheesequake[6]	12	11	0.20	56	0.20	21
North Carolina, Roanoke Island[2,3]	71	35	0.08	56	0.08	26
Florida, Little Manatee River[7]	37	15	0.07	80	0.07	32
Connecticut, East River Marsh[8]	84	28	0.16	57	0.16	19
Massachusetts, Wood Island[2]	14	4	0.06	74	0.06	22
Massachusetts, Barnstable[9–11]	7	2	0.62	335	0.62	239
Connecticut, Barn Island[12]	11	37	0.08	87	0.08	36
Florida, Nassau[13]	39	12	0.07	67	0.07	17
Nova Scotia, Chezzetcook Inlet[12]	48	50	0.06	32	0.06	12
Newfoundland, Big River Marsh[14]	86	24	0.12	52	0.08	25
Iceland, Vioarholmi[15]	50	36	0.18	67	0.18	11
Quebec, Saint-Simeon[16]	67	34	0.16	56	0.16	14
Newfoundland, Placentia[14]	35	16	0.08	96	0.12	30
Denmark, Ho Bugt[17,18]	19	1	0.24	92	0.22	113
Scotland, Loch Laxford[19]	30	33	0.35	129	0.35	12
Scotland, Kyle of Tongue[19]	21	18	0.35	95	0.35	32
Greenland, Aasiaat[20]	13	3	0.15	65	0.13	92
Greenland, Sisimiut[20]	14	3	0.12	84	0.12	89

Supplementary Table 3. Combined nonlinear regional and local 1700–2000 CE rate and the probability that the rate is positive.

Site	Nonlinear regional and local 1700–2000 CE rate (mm/yr)	Probability that 1700–2000 CE rate is positive
New York, Pelham Bay	0.26 ± 0.12	0.985
North Carolina, Cedar Island	0.33 ± 0.12	0.997
New Jersey, Cape May Courthouse	0.39 ± 0.13	0.999
New Jersey, Cheesequake	0.26 ± 0.15	0.960
North Carolina, Roanoke Island	0.22 ± 0.12	0.966
Florida, Little Manatee River	0.31 ± 0.13	0.994
Connecticut, East River Marsh	0.15 ± 0.14	0.863
Massachusetts, Wood Island	0.15 ± 0.14	0.868
Massachusetts, Barnstable	0.12 ± 0.15	0.783
Connecticut, Barn Island	0.16 ± 0.13	0.891
Florida, Nassau	0.09 ± 0.13	0.745
Nova Scotia, Chezzetcook Inlet	-0.14 ± 0.11	0.117
Newfoundland, Big River Marsh	-0.01 ± 0.12	0.466
Iceland, Vioarholmi	-0.05 ± 0.15	0.358
Quebec, Saint-Simeon	-0.13 ± 0.14	0.176
Newfoundland, Placentia	-0.10 ± 0.13	0.232
Denmark, Ho Bugt	-0.15 ± 0.16	0.172
Scotland, Loch Laxford	-0.21 ± 0.15	0.090
Scotland, Kyle of Tongue	-0.17 ± 0.15	0.141
Greenland, Aasiaat	-0.19 ± 0.16	0.125
Greenland, Sisimiut	-0.17 ± 0.16	0.145

Supplementary Table 4. Comparison of time of emergence using 40-year, 60-year, and 80-year average rates.

	Time of emergence		
Site	40-year rates	60-year rates	80-year rates
Global sea level	1870 (1835-1879)	1863 (1825–1873)	1855 (1818-1867)
New York, Pelham Bay	1983 (1856-NYE)	1872 (1840–1906)	1865 (1827-1876)
North Carolina, Cedar Island	1916 (1856-1965)	1874 (1837–1894)	1861 (1817-1875)
New Jersey, Cape May Courthouse	1914 (1856-1951)	1880 (1837–1898)	1866 (1815-1878)
New Jersey, Cheesequake	1938 (1862-1973)	1889 (1846–1906)	1872 (1830-1885)
North Carolina, Roanoke Island	1961 (1869-1973)	1894 (1842–1910)	1872 (1823-1889)
Florida, Little Manatee River	1931 (1871-1956)	1897 (1838–1912)	1869 (1812-1891)
Connecticut, East River Marsh	1940 (1873-1975)	1897 (1855–1911)	1878 (1843-1892)
Massachusetts, Wood Island	1971 (1883-NYE)	1906 (1856–1927)	1883 (1843-1899)
Massachusetts, Barnstable	1962 (1885-NYE)	1908 (1859–1926)	1887 (1847-1900)
Connecticut, Barn Island	1942 (1887-1973)	1908 (1860–1926)	1889 (1850-1902)
Florida, Nassau	1950 (1908-1974)	1919 (1864–1938)	1897 (1847-1917)
Nova Scotia, Chezzetcook Inlet	1952 (1913-NYE)	1930 (1895–1944)	1914 (1883-1926)
Newfoundland, Big River Marsh	1974 (1923-NYE)	1942 (1900–1959)	1925 (1878-1941)
Iceland, Vioarholmi	1980 (1915-NYE)	1947 (1882–NYE)	1918 (1862-1937)
Quebec, Saint-Simeon	NYE (1931-NYE)	1949 (1903–NYE)	1930 (1881-1953)
Newfoundland, Placentia	1976 (1930-NYE)	1951 (1908–1967)	1940 (1887-1954)
Denmark, Ho Bugt	NYE (1918-NYE)	1957 (1889–NYE)	1922 (1876-1945)
Scotland, Loch Laxford	NYE (1919-NYE)	1958 (1891–NYE)	1928 (1878-1948)
Scotland, Kyle of Tongue	NYE (1920-NYE)	1962 (1890–NYE)	1928 (1876-1951)
Greenland, Aasiaat	NYE (1933-NYE)	1963 (1902–NYE)	1936 (1884-1959)
Greenland, Sisimiut	NYE (1934-NYE)	1964 (1901–NYE)	1936 (1884-1958)

Time of emergence is the year when the probability reaches 0.90 with an uncertainty range of a lower bound when the probability reaches 0.66 and an upper bound when the probability reaches 0.95. NYE = not yet emerged.

Supplementary Table 5. Comparison of time of emergence using a background reference period of 0–1700 CE or 0–1400 CE.

Site	Time of emergence (0–1700 reference period)	Time of emergence (0–1400 reference period)
Global sea level	1863 (1825–1873)	1862 (1822–1873)
New York, Pelham Bay	1872 (1840–1906)	1868 (1836–1898)
North Carolina, Cedar Island	1874 (1837–1894)	1872 (1836–1894)
New Jersey, Cape May Courthouse	1880 (1837–1898)	1880 (1837–1898)
New Jersey, Cheesequake	1889 (1846–1906)	1888 (1846–1906)
North Carolina, Roanoke Island	1894 (1842–1910)	1895 (1842–1913)
Florida, Little Manatee River	1897 (1838–1912)	1892 (1831–1908)
Connecticut, East River Marsh	1897 (1855–1911)	1897 (1855–1911)
Massachusetts, Wood Island	1906 (1856–1927)	1906 (1855–1926)
Massachusetts, Barnstable	1908 (1859–1926)	1907 (1858–1925)
Connecticut, Barn Island	1908 (1860–1926)	1907 (1859–1926)
Florida, Nassau	1919 (1864–1938)	1919 (1864–1938)
Nova Scotia, Chezzetcook Inlet	1930 (1895–1944)	1931 (1895–1944)
Null site	1933 (1877–1961)	1932 (1876–1960)
Newfoundland, Big River Marsh	1942 (1900–1959)	1940 (1895–1954)
Iceland, Vioarholmi	1947 (1882–NYE)	1947 (1883–NYE)
Quebec, Saint-Simeon	1949 (1903–NYE)	1947 (1901–NYE)
Newfoundland, Placentia	1951 (1908–1967)	1948 (1900–1964)
Denmark, Ho Bugt	1957 (1889–NYE)	1958 (1889–NYE)
Scotland, Loch Laxford	1958 (1891–NYE)	1959 (1891–NYE)
Scotland, Kyle of Tongue	1962 (1890–NYE)	1963 (1890–NYE)
Greenland, Aasiaat	1963 (1902–NYE)	1958 (1897–NYE)
Greenland, Sisimiut	1964 (1901–NYE)	1958 (1897–NYE)

Time of emergence is the year when the probability reaches 0.90 with an uncertainty range of a lower bound when the probability reaches 0.66 and an upper bound when the probability reaches 0.95. NYE = not yet emerged.

Supplementary Table 6. Comparison of global time of emergence using different datasets.

Dataset	Time of emergence
Kopp et al. [21] database	1876 (1847-1885)
Kemp et al. [14] database	1878 (1841-1887)
This study: all data	1863 (1825–1873)
Only North Atlantic sites	1859 (1790-1877)
All data excluding North Atlantic sites	1858 (1804-1874)
All data excluding western North Atlantic sites	1887 (1854-1898)

Time of emergence is the year when the probability reaches 0.90 with an uncertainty range of a lower bound when the probability reaches 0.66 and an upper bound when the probability reaches 0.95.

Supplementary Table 7. Time of emergence for European sites with reduced uncertainties.

Site	Time of emergence	Time of emergence with reduced proxy data errors
Iceland, Vioarholmi	1947 (1882–NYE)	1942 (1885–NYE)
Denmark, Ho Bugt	1957 (1889–NYE)	NYE (1902–NYE)
Scotland, Loch Laxford	1958 (1891–NYE)	1968 (1922–NYE)
Scotland, Kyle of Tongue	1962 (1890–NYE)	1968 (1912–NYE)
Greenland, Aasiaat	1963 (1902–NYE)	1966 (1908–NYE)
Greenland, Sisimiut	1964 (1901–NYE)	1969 (1910–NYE)

Proxy data relative sea level and chronological uncertainties for European sites were reduced to be comparable to the uncertainties of proxy data on the North American coast. Specifically, the 1σ relative sea level uncertainty was set to a maximum of 0.1 m and the 2σ chronological uncertainty was set to a maximum of 100 years. Time of emergence is the year when the probability reaches 0.90 with an uncertainty range of a lower bound when the probability reaches 0.66 and an upper bound when the probability reaches 0.95. NYE = not yet emerged.

References

1. Kemp, A. C. *et al.* Relative sea-level trends in New York City during the past 1500 years. *The Holocene* **27**, 1169–1186 (2017b).
2. Kemp, A. C. *et al.* Climate related sea-level variations over the past two millennia. *Proc. Natl. Acad. Sci.* 6 (2011).
3. Kemp, A. C. *et al.* Extended late Holocene relative sea-level histories for North Carolina, USA. *Quat. Sci. Rev.* **160**, 13–30 (2017).
4. Kemp, A. C. *et al.* Sea-level change during the last 2500 years in New Jersey, USA. *Quat. Sci. Rev.* **81**, 90–104 (2013).
5. Cahill, N., Kemp, A. C., Horton, B. P. & Parnell, A. C. A Bayesian hierarchical model for reconstructing relative sea level: from raw data to rates of change. *Clim. Past* **12**, 525–542 (2016).
6. Walker, J. S. *et al.* Common Era sea-level budgets along the U.S. Atlantic coast. *Nat. Commun.* (2021).
7. Gerlach, M. J. *et al.* Reconstructing Common Era relative sea-level change on the Gulf Coast of Florida. *Mar. Geol.* **390**, 254–269 (2017).
8. Kemp, A. C. *et al.* Relative sea-level change in Connecticut (USA) during the last 2200 yrs. *Earth Planet. Sci. Lett.* **428**, 217–229 (2015).
9. Redfield, A. C. & Rubin, M. The age of salt marsh peat and its relation to recent changes in sea level at Barnstable, Massachusetts. *Proc. Natl. Acad. Sci. U. S. A.* **48**, 1728–1735 (1962).
10. Stuiver, M., Deevey, E. S. & Rouse, I. Yale Natural Radiocarbon Measurements VIII. *Radiocarbon* **5**, 312–341 (1963).

11. Engelhart, S. E. & Horton, B. P. Holocene sea level database for the Atlantic coast of the United States. *Quat. Sci. Rev.* **54**, 12–25 (2012).

12. Gehrels, W. R. *et al.* A Preindustrial Sea-Level Rise Hotspot Along the Atlantic Coast of North America. *Geophys. Res. Lett.* **47**, e2019GL085814 (2020).

13. Kemp, A. C. *et al.* Late Holocene sea- and land-level change on the U.S. southeastern Atlantic coast. *Mar. Geol.* **357**, 90–100 (2014).

14. Kemp, A. C. *et al.* Relative sea-level change in Newfoundland, Canada during the past ~3000 years. *Quat. Sci. Rev.* **201**, 89–110 (2018).

15. Gehrels, W. R. *et al.* Rapid sea-level rise in the North Atlantic Ocean since the first half of the nineteenth century. *The Holocene* **16**, 949–965 (2006).

16. Barnett, R. L. *et al.* Late Holocene sea-level changes in eastern Québec and potential drivers. *Quat. Sci. Rev.* **203**, 151–169 (2019).

17. Gehrels, W. R. *et al.* Late Holocene sea-level changes and isostasy in western Denmark. *Quat. Res.* **66**, 288–302 (2006).

18. Szkornik, K., Gehrels, W. R. & Murray, A. S. Aeolian sand movement and relative sea-level rise in Ho Bugt, western Denmark, during the `Little Ice Age'. *The Holocene* **18**, 951–965 (2008).

19. Barlow, N. L. M. *et al.* Salt-marsh reconstructions of relative sea-level change in the North Atlantic during the last 2000 years. *Quat. Sci. Rev.* **99**, 1–16 (2014).

20. Long, A. J. *et al.* Relative sea-level change in Greenland during the last 700yrs and ice sheet response to the Little Ice Age. *Earth Planet. Sci. Lett.* **315–316**, 76–85 (2012).

21. Kopp, R. E. *et al.* Temperature-driven global sea-level variability in the Common Era. *Proc. Natl. Acad. Sci.* **113**, E1434–E1441 (2016).
